# Describing nearly two decades of Chagas disease in Germany and the lessons learned: a retrospective study on screening, detection, diagnosis, and treatment of *Trypanosoma cruzi* infection from 2000 – 2018

**DOI:** 10.1186/s12879-020-05600-8

**Published:** 2020-12-03

**Authors:** Jessica Michelle Guggenbühl Noller, Guenter Froeschl, Philip Eisermann, Johannes Jochum, Stefanie Theuring, Ingrid Reiter-Owona, Alfred Lennart Bissinger, Michael Hoelscher, Abhishek Bakuli, Franz-Josef Falkner von Sonnenburg, Camilla Rothe, Gisela Bretzel, Pedro Albajar-Viñas, Lise Grout, Michael Pritsch

**Affiliations:** 1 Division of Infectious Diseases and Tropical Medicine, University Hospital, LMU Munich, Munich, Germany; 2 Center for International Health, University Hospital, LMU Munich, Munich, Germany; 3 National Reference Centre for Tropical Pathogens, Bernhard Nocht Institute for Tropical Medicine, Hamburg, Germany; 4 Department of Tropical Medicine, Bernhard Nocht Institute for Tropical Medicine & I. Department of Medicine, University Medical Centre Hamburg-Eppendorf, Hamburg, Germany; 5 Institute of Tropical Medicine and International Health, Charité –Universitätsmedizin Berlin, corporate member of Freie Universität Berlin, Humboldt-Universität Berlin, and Berlin Institute of Health, Berlin, Germany; 6 Institute of Medical Microbiology, Immunology and Parasitology, University Hospital Bonn, Bonn, Germany; 7 Institute of Tropical Medicine, Medical Department, University Hospital Tübingen, Tübingen, Germany; 8 German Center for Infection Research (DZIF), Partner Site Munich, Munich, Germany; 9 Department of Control of Neglected Tropical Diseases, World Health Organization, Geneva, Switzerland

**Keywords:** *Trypanosoma cruzi*, Chagas, Germany, Screening, Diagnosis, Treatment

## Abstract

**Background:**

The highly complex and largely neglected Chagas disease (CD) has become a global health problem due to population movements between Latin America and non-endemic countries, as well as non-vectorial transmission routes. Data on CD testing and treatment from routine patient care in Germany of almost two decades was collected and analysed.

**Methods:**

German laboratories offering diagnostics for chronic *Trypanosoma cruzi* (*T. cruzi*) infection in routine patient care were identified. All retrievable data on tests performed during the years of 2000–2018 were analysed. Additional clinical information regarding patients diagnosed with CD was collected through questionnaires.

**Results:**

Five German laboratories with diagnostics for *T. cruzi* infection in routine patient care were identified. Centres in Hamburg and Munich offered two independent serological tests to confirm the CD diagnosis, as recommended by WHO during the entire time period 2000–2018. Overall, a total of *n* = 10,728 independent tests involving *n* = 5991 individuals were identified with a progressive increase in testing rates over time, only *n* = 130 (16.0%) of the tested individuals with known nationality came from CD endemic countries. Of all test units conducted at the included institutes, a total of *n* = 347/10,728 (3.2%) tests on CD were positive, of which *n* = 200/347 (57.6%) were ELISA, *n* = 133/347 (38.3%) IFT, *n* = 10/347 (2.9%) PCR, and *n* = 4/347 (1.2%) RDT. Of the *n* = 5991 individuals only *n* = 81 (1.4%) with chronic infection were identified, *n* = 52 females and *n* = 28 males. Additional clinical information could only be collected from *n* = 47.

**Conclusion:**

The results of this study give insight into the deployment of screening, detection, diagnosis, and treatment of *T. cruzi* over the last two decades in Germany and existing deficits therein; the creation of guidelines for Germany could be a step forward to improve the existing gaps.

**Supplementary Information:**

The online version contains supplementary material available at 10.1186/s12879-020-05600-8.

## Background

Chagas disease (CD) —a highly complex parasitic disease caused by *Trypanosoma cruzi* (*T. cruzi*) infection— can lead to chronic morbidity, complications, and premature death with all associated socio-economic effects. Estimates by WHO state that there are between 6 and 7 million infected individuals world-wide, mostly in Latin American (LA) countries [1]. The initial acute phase of CD is followed by chronic infection. After a variable period of latency, about 30–40% of people develop clinical alterations that can be both symptomatic as well as asymptomatic. Vectorial transmission accounts for most infections in LA. However, population movements between endemic and non-endemic areas as well as non-vectorial transmission routes expanded the geographical distribution of CD, with some 400,000 infected persons living all over the world [2].

The initial period of reported CD infections in Europe started in 1981 with the first published report: A probable case of congenital transmission in Romania was described, that likely took place in 1975 [3]. Then a report in 1990 described the first known *T. cruzi* infection in Switzerland, dated in 1979 [4]. A growing number of publications on *T. cruzi* infections in Europe [5] during the second period were sparked by increasing migration from LA to Europe, specifically to Spain between 1996 and 2001 [6]. The third period began in 2007 when the WHO and PAHO convoked a meeting and the presence of CD in non-endemic countries was officially recognized. In the following year, a meeting in Spain was used to assess the status of CD in Europe and develop strategies for screening and detection, as well as diagnosis. A statement during the 6th European Congress on Tropical Medicine and International Health in Verona 2009 considered the evidence sufficient in order to officially recognize CD as a public health concern in Europe [7].

In a study performed in 2015, Requena-Mendez and colleagues estimated an overall prevalence of 4.2% among the adult LA migrant population in Europe [8] and that some 94–96% of those infected likely have not been diagnosed [9]. This lack of reliable epidemiological data impedes adequate screening, detection, diagnosis, and eventually treatment. Furthermore, knowledge on CD is very limited among physicians, although adequate diagnosis and treatment can potentially cure patients, as well as prevent chronic morbidity, complications, sudden cardiac death, and non-vectorial transmission [1]. Thus, screening of LA migrants for CD in Europe can be considered cost-effective [10].

A total of *n* = 127,615 immigrants from endemic countries were registered in Germany in 2018 [11], not taking into account undocumented migrants or migrants with European citizenship. Many experience difficulties to approach the German healthcare system due to legal, economic, and linguistic problems among others [12]. The first German epidemiologic study on CD was published in 1997, in which *n* = 100 LA migrants were screened for CD and *n* = 2 were diagnosed with CD [13]. Since then, there has been a lack of published epidemiological data regarding CD in Germany. In a study where the literature and official data of six different European countries, was analysed, the results showed that by 2009, only 4290 of the 68,000–122,000 CD cases that were expected in Europe were diagnosed and reported. In Germany, the observed rate was only 0.002%, which according to the expected prevalence of CD indicated that 99% of the cases in the immigrant population were undiagnosed, based on the number of LA migrants [9]. Only one additional study performed —in Munich, Germany— aimed to find the prevalence of CD in a specific community of LA migrants between 2013 and 2014, within the city of Munich. This study showed that four of the 43 participants (9.3%) tested positive for CD. Nonetheless, there is limited supplementary information regarding the epidemiological prevalence of CD in Germany, including the geographical distribution and the specification of different cases —for example, infections through congenital transmission. Additionally, German guidelines and policies concerning CD are missing [12]: Presently, there are no screening measures for non-vectorial transmission; such as screening of blood products or pregnant women originating from endemic countries [9, 12, 14]. In order to break these identified barriers for access to adequate diagnosis and treatment, a multidimensional approach is needed, far beyond a biomedical one [15].

In order to diagnose CD it is currently recommended to perform two different serological tests [16]. Enzyme-linked immunosorbent assays (ELISA), immunofluorescence tests (IFT), as well as rapid diagnostic tests (RDT) –among others– are currently commercially available in Germany. Polymerase chain reaction (PCR) can also be used to monitor patients in certain situations, making it an effective alternative [17]. Additionally, benznidazole (BNZ) and nifurtimox are used for etiologic treatment and despite being included in the *Model List of Essential Medicines* [18], are neither licensed nor available on the market in Germany and obtainable only through WHO donations [12].

Due to the aforementioned lack of information, education, awareness, and severe structural barriers, up to 99% of CD cases might not be diagnosed in Germany and only a fraction of those diagnosed might receive adequate care [9]. The objective herein is to describe retrospectively available data on testing and treatment of CD in Germany during nearly two decades. This will hopefully fuel discussion on this highly neglected tropical disease and lay a foundation for the development of German guidelines as well as improving policies concerning all aspects of CD.

## Methods

### Study design

**Fig. 1:**
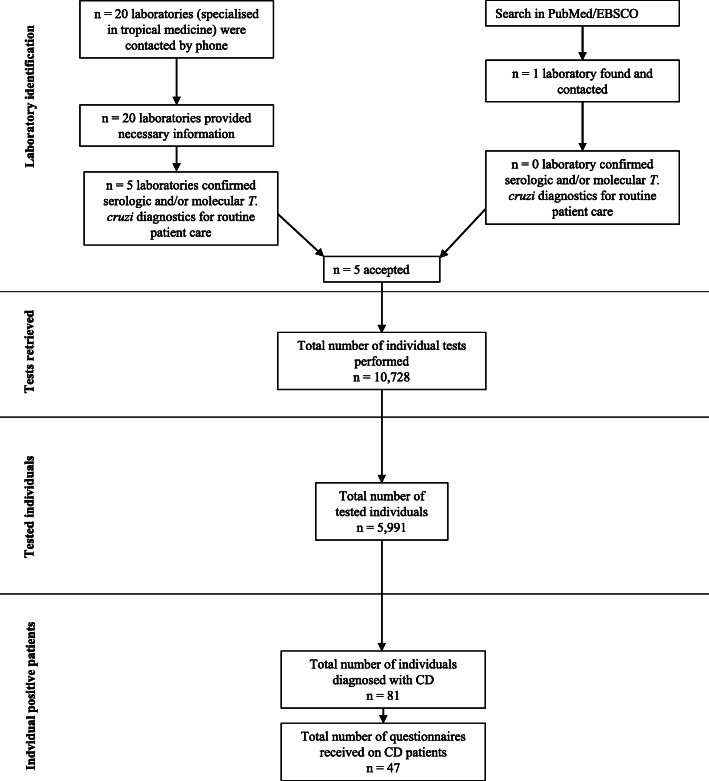
Methodological flow diagram

The study protocol was approved by the Institutional Review Board at the Ludwig-Maximilians-Universität in Munich, Germany (opinion date 21 December 2018, number 18–889) and adhered to the most recent version of the Declaration of Helsinki. It is a retrospective descriptive analysis of retrievable data on CD testing and cases during routine patient care in Germany and the years of 2000–2018. Firstly, all laboratories offering quality-assured serological and/or molecular *T. cruzi* diagnostics for routine patient care in Germany were identified by considering two sources of information: (i) all centres specialised in tropical medicine listed by the German Society for Tropical Medicine and Global Health (DTG) on their webpage [19] were contacted and asked about laboratories offering quality-assured CD diagnostics for routine patient care in Germany; and (ii) an extensive search for German laboratories offering CD diagnostics in patient care was carried out in PubMed and EBSCO (see also Fig. 1). After establishing contact with the respective laboratories, information on existing CD diagnostics as well as consent to participate in this study were sought out.

### Data collection and analysis

Laboratories were asked to provide all available data on CD diagnostics for the years 2000–2018. Data on type, laboratory number, year, and result of each individual test performed as well as the demographic data (age, sex, nationality) of the tested individual were collected in a pseudonymised way, by extracting available databases. The diagnosis of CD was considered to be established if (i) two different serological tests were positive, or (ii) a CD-specific PCR was positive, or (iii) a patient had previously been diagnosed elsewhere (e.g. abroad). In some cases, the second serological test for confirmation was conducted outside the reporting laboratories by forwarding samples to an external facility. For all identified CD cases, the responsible physicians were asked to fill in a questionnaire, containing more detailed questions about medical data (see S1 File).

Data quality was analysed throughout the collection phase and pseudonymised data were entered in a password secured Excel database. Duplicates were identified by using the laboratory ID, gender, and date of birth of the patients. Once all the data were collected and data quality was assured, the data were irreversibly anonymised and analysed. Descriptive statistical analyses were performed using the statistical computing software CRAN R 3.6.0 [20] and the corresponding libraries ggplot2 for graphics, reshape2 for tabulations, and FSA for the tables with summary statistics. Continuous data means and standard deviations were calculated. Logistic regression was performed to obtain the Odds Ratio (OR) and 95% confidence interval (95% CI) estimates to evaluate risk factors {(Sex- (Male, Female, Missing), Age (continuous), Nationality (Brazilian/Bolivian, Others, Missing)} for having positive test results vs. negative test results within the data observed for Munich (the subgroup from Munich was chosen due to availability of data for the variables to be included in the regression analysis); *p* < 0.05 was considered statistically significant. Due to the high proportion of missing data for the risk factor categories (like nationality and sex), and very small count of positives we used Fischer Exact tests to obtain updated OR and 95% CI leaving out the missing data category.

Double nationality was handled by acknowledging only the endemic country, as all cases consisted of German nationality plus one of a CD endemic country.

## Results

### German laboratories with CD diagnostics for patient care during 2000–2018

A total of five German laboratories were identified as having offered serological and/or molecular CD tests for routine patient care during 2000–2018 (Table 1). All identified laboratories agreed to participate in this study (see Fig. 1). All belong to medical centres specialised in tropical medicine and tests offered were quality controlled according to the respective German regulations and existing laboratory standards for routine patient care. Only two centres —one in Hamburg, the other in Munich— offered the internationally-required two independent serological assays for CD diagnosis, throughout the years of 2000–2018 as required by international standards [16]. The range of diagnostics available in Germany changed over the years 2000–2018 (Table 1). A more detailed description on test types as well as procedures used can be found in Supplement S2.

**Table 1:** German centres with CD diagnostics for routine patient care during 2000–2018

Center	City	Type of test	Test availability (years)	Number of retrieved tests	Data availability (years)
Institute of Tropical Medicine and International Health, Charité – Universitätsmedizin in Berlin	Berlin	in-house IFT	2000–2010	204	2002–2018
		in-house ELISA	2000–2018	830	
Institute of Medical Microbiology, Immunology and Parasitology, University Hospital Bonn	Bonn	in-house IFT	2000–2010	201	2004–2018
		commercial ELISA	2011–2018	190	
		commercial RDT	2000–2010	116	
Bernhard Nocht Institute for Tropical Medicine	Hamburg	in-house IFT	2000–2018	2641	2000–2018^a^
		in-house ELISA	2000–2018	2654	
		PCR^b^	2000–2018	200	
Division of Infectious Diseases and Tropical Medicine, University Hospital, LMU Munich	Munich	in-house IFT	2000–2018	1670	2000–2018
		in-house ELISA	2000–2018	1668	
		PCR^b^	2013–2018	35	
Institute of Tropical Medicine, Medical Department, University Hospital Tübingen	Tuebingen	in-house IFT	2009–2018	319	2009–2018
			Total	10,728	

*CD* Chagas disease, *ELISA* Enzyme-linked immuno-sorbent assay, *IFT* Immunofluorescence test, *PCR* Polymerase chain reaction, *RDT* Rapid diagnostic test

^a^Individual positive tests 2000–2018 and negative tests 2010–2018 could be retrieved

^b^Hamburg offered a conventional PCR that was followed up by sequencing of the PCR product if positive, whereas Munich performed a conventional PCR that was followed up with an independent commercially available qPCR if positive (for details please see Supplement S2)

### Number of diagnostic tests on CD

**Fig. 2:**
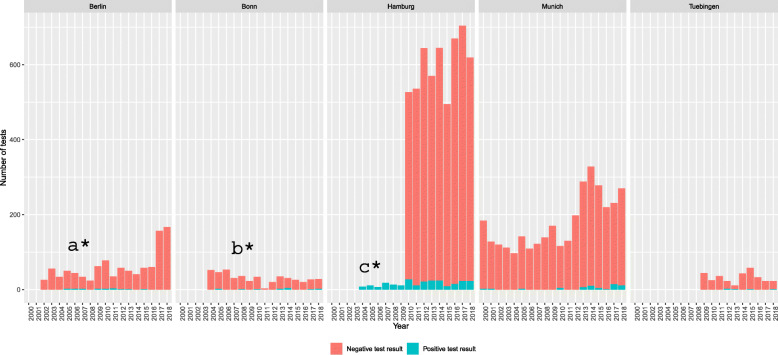
Retrieved tests for *T. cruzi* infection per centre in the years 2000-2018 in Germany. Berlin performed in-house ELISA (2000-2018) and in-house IFT (2000-2010). Bonn performed commercial ELISA (2011-2018), in-house IFT (2000-2010), and commercially available RDT (2000-2010); Hamburg performed in-house ELISA, in-house IFT, and PCR (2000-2018); Munich performed in-house ELISA (2000-2018), in-house IFT (2000-2018), and PCR (2013-2018); Tübingen in-house IFT (2009-2018); ^a*^Data could be retrieved for the years 2002-2018; ^b*^Data could be retrieved for the years 2004-2018; ^c*^Only individual positive tests from 2000-2009 could be retrieved

A total of *n* = 10,728 individual tests (ELISA, IFT, (q) PCR, and RDT) on *n* = 5991 patients could be included as depicted in Table 1 as well as Fig. 1. The annual frequency of tests and patients increased over time (Fig. 2). Data on a total of *n* = 2479 negatively tested individuals in Hamburg during 2000–2009 could only be retrieved in aggregated form without demographic details. Thus, these were not included in Table 1 and subsequent analyses.

### Data on all tested individuals

In Table 2, demographic data on all tested individuals is presented as available. Mean age was 39.1 (SD 16.7) years at the time of testing and roughly the same proportion of females and males were tested, with females being slightly younger at the time of testing. A total of *n* = 1695 (73.3%) tested females were of childbearing age (range 15–49 years). Information on nationality was only available in 13.6% of all tested individuals. Of these, *n* = 130 individuals (16.0%) were from 14 endemic countries, with Brazil (*n* = 36), Bolivia (*n* = 31), Colombia (*n* = 13), Peru (*n* = 12), Ecuador (*n* = 8), Argentina (*n* = 7), and Venezuela (*n* = 6) being the most frequent nationalities mentioned.

**Table 2:** Demographic data of individuals tested for *Trypanosoma cruzi* infection in Germany and the years 2000–2018

Sex	Female *n* = 2389/5991 39.9%	Male *n* = 2365/5991 39.5%	Unknown *n* = 1237/5991 20.6%	All *n* = 5991 100.0%
Age at time of testing	Data on age available on 2312/2389 (96.8%) *n* = 2312	Data on age available on 2322/2365 (98.2%) *n* = 2322	Data on age available on 277/1237 (22.4%) *n* = 277	Data on age available on 4911/5991 (82.0%) *n* = 4911
Mean (SD)	37.4 years (SD = 16.0)	41.2 years (SD = 16.6)	35.4 years (SD = 21.3)	39.1 years (SD = 16.7)
≤14	78 (3.4%)	87 (3.7%)	67 (24.2%)	232 (4·7%)
15–49	1695 (73.3%)	1474 (63.5%)	146 (52.7%)	3315 (67.5%)
≥50	539 (23.3%)	761 (32.8%)	64 (23.1%)	1364 (27.8%)
Nationality	Data on nationality available on 417/2389 (17.5%) *n* = 417	Data on nationality available on 378/2365 (16.0%) *n* = 378	Data on nationality available on 19/1237 (1.5%) *n* = 19	Data on nationality available on 814/5991 (13.6%) *n* = 814
Argentina	7 (1.7%)	0	0	7 (0.9%)
Bolivia	18 (4.3%)	13 (3.4%)	0	31 (3.8%)
Brazil	24 (5.8%)	9 (2.4%)	3 (15.8%)	36 (4.4%)
Colombia	11 (2.6%)	2 (0.5%)	0	13 (1.6%)
Costa Rica	1 (0.2%)	0	0	1 (0.1%)
Ecuador	4 (1.0%)	4 (1.1%)	0	8 (1.0%)
El Salvador	1 (0.2%)	0	0	1 (0.1%)
Guatemala	1 (0.2%)	0	0	1 (0.1%)
Mexico	5 (1.2%)	0	0	5 (0.6%)
Nicaragua	1 (0.2%)	0	0	1 (0.1%)
Panama	0	1 (0.3%)	0	1 (0.1%)
Paraguay	5 (1.2%)	2 (0.5%)	0	7 (0.9%)
Peru	6 (1.4%)	6 (1.6%)	0	12 (1.5%)
Venezuela	4 (1.0%)	1 (0.3%)	1 (5.3%)	6 (0.7%)
Germany	297 (71.2%)	318 (84.1%)	12 (63.2%)	627 (77.0%)
Other non-endemic countries	32 (7.7%)	22 (5.8%)	3 (15.8%)	57 (7.0%)

SD Standard deviation

#### Data on patients diagnosed with CD

Of all test units conducted at the included institutes, a total of *n* = 347/10,728 (3.2%) tests on CD were positive, of which there were *n* = 200/347 (57.6%) ELISA, *n* = 133/347 (38.3%) IFT, *n* = 10/347 (2.9%) PCR, and *n* = 4/347 (1.2%) RDT. From an individual patient perspective, of all *n* = 5991 included individuals, a total of *n* = 81 (1.4%) CD patients could be identified (Table 3). Of the *n* = 81 identified CD patients all had positive serology, in addition in 10 patients there was an additional positive PCR result.

**Table 3:** Characteristics of patients diagnosed with CD, Germany, 2000–2018

Sex^a^	Female *n* = 52/81 (64.2%)	Male *n* = 28/81 (34.6%)	All *n* = 81 (100.0%)
Age in years at the time of testing			
Mean (SD)	43.3 years (SD = 3.8)	46.1 years (SD = 6.9)	44.0 years (SD = 3.4)
≤ 14	0	1 (3.6%)	1 (1.2%)
15–49	35 (67.3%)	16 (57.1%)	51 (63.0%)
≥ 50	17 (32.7%)	11 (39.3%)	28 (34.6%)
NA	0	0	1 (1.2%)
Nationality	Available on *n* = 26/52 (50.0%)	Available on *n* = 9/28 (32.1%)	Available on *n* = 35/81 (43.2%)
Bolivia	13 (50.0%)	7 (77.8%)	20 (57.1%)
Germany	0	2 (22.2%)	2 (5.7%)
Brazil	5 (19.2%)	0	5 (14.3%)
Paraguay	2 (7.7%)	0	2 (5.7%)
Argentina	3 (11.5%)	0	3 (8.6%)
Peru	2 (7.7%)	0	2 (5.7%)
South America	1 (3.8%)	0	1 (2.9%)
Reason for testing	Available on *n* = 33/52 (63.5%)	Available on *n* = 12/28 (42.9%)	Available on *n* = 45/81 (55.6%)
Known CD	17 (51.5%)	7 (58.3%)	24 (53.3%)
Endemic national	7 (21.2%)	2 (16.7%)	9 (20.0%)
Other	9 (27.3%)	3 (25.0%)	12 (26.7%)
Symptoms	Available on *n* = 23/52 (44.2%)	Available on *n* = 9/28 (32.1%)	Available on *n* = 32/81 (39.5%)
Fatigue	0	2 (22.2%)	2 (6.3%)
Gastrointestinal	9 (39.1%)	2 (22.2%)	11 (34.4%)
Cardiac	3 (13.0%)	0	3 (9.4%)
Other	1 (4.3%)	2 (22.2%)	3 (9.4%)
No symptoms	10 (43.5%)	3 (33.3%)	13 (40.6%)
Initial diagnosis of CD	Available on *n* = 30/52 (57.7%)	Available on *n* = 13/28 (46.4%)	Available on *n* = 43/81 (53.1%)
Yes	10 (33.3%)	5 (38.5%)	15 (34.9%)
No	20 (66.7%)	8 (61.5%)	28 (65.1%)
Echocardiography	Available on *n* = 18/52 (34.6%)	Available on *n* = 5/28 (17.9%)	Available on *n* = 23/81 (28.4%)
Normal	16 (88.9%)	3 (60.0%)	19 (82.6%)
Abnormal	0	1 (20.0%)	1 (4.3%)
Not performed	2 (11.1%)	1 (20.0%)	3 (13.0%)
Electrocardiography	Available on *n* = 18/52 (34.6%)	Available on *n* = 9/28 (32.1%)	Available on *n* = 27/81 (33.3%)
Normal	16 (88.9%)	6 (66.7%)	22 (81.5%)
Abnormal	2 (11.1%)	2 (22.2%)	4 (14.8%)
Not performed	0	1 (11.1%)	1 (3.7%)
Etiologic treatment	Available on *n* = 22/52 (42.3%)	Available on *n* = 7/28 (25.0%)	Available on *n* = 29/81 (35.8%)
Benznidazole	8 (36.4%)	3 (42.9%)	11 (37.9%)
Nifurtimox	2 (9.1%)	0	2 (6.9%)
No treatment	12 (54.5%)	4 (57.1%)	16 (55.2%)
Treatment course	Available on *n* = 8/10 (80.0%)	Available on *n* = 1/3 treatments (33.3%)	Available on *n* = 9/13 treatments (69.2%)
Completed treatment	5 (62.5%)	0	5 (55.6%)
Stopped treatment	3 (37.5%)	1 (100%)	4 (44.4%)
Side effects	Available on *n* = 3/10 treatments (30.0%)	Available on *n* = 1/3 treatments (33.3%)	Available on *n* = 4/13 treatments (30.8%)
Yes	3 (100%)	1 (100%)	4 (100%)
No	0	0	0
Reason for no treatment	Available on *n* = 11/12 (91.7%)	Available on n = 4/4 (100%)	Available on *n* = 15/16 (93.8%)
Recommendations-based reason	5 (45.5%)	2 (50.0%)	7 (46.7%)
Non-recommendations-based reason	6 (54.5%)	2 (50.0%)	8 (53.3%)
Follow-up	Available on *n* = 13/52 (25.0%)	Available on *n* = 4/28 (14.3%)	Available on *n* = 17/81 (21.0%)
> 12 months	7 (53.8%)	3 (75.0%)	10 (58.8%)
≤ 12 months	4 (30.8%)	1 (25.0%)	5 (29.4%)
No follow-up	2 (15.4%)	0	2 (11.8%)

^a^in one CD case none of the data was available; *CD* Chagas disease, *NA* Not available, *SD* Standard deviation

For all *n* = 81 CD patients identified, treating physicians were asked to provide additional information via questionnaires. A total of *n* = 47 (58.0%) were returned. Based on the returned questionnaire data, *n* = 15/43 (34.9%) CD cases were diagnosed for the first time. Of all *n* = 29 CD patients for whom information on treatment was provided, a total of *n* = 16 (55.2%) were identified not to have received etiologic treatment. For *n* = 7 patients without etiologic treatment (46.7%), physicians stated justifications that were in accordance with current recommendations and included advanced age, refusal by patient, and severe organ dysfunction. In *n* = 8 patients without etiologic treatment (53.3%), physicians stated justifications that were not in accordance with current recommendations and included “no symptoms by the patient,” “no sign of active CD,” “no proof of chronic CD,” “negative PCR result,” and “chronic case of CD.” A total of *n* = 35/80 (43.8%) patients were females of childbearing age and the most prevalent nationality among those with respective information available was Bolivian *n* = 20/35 (57.1%).

For the subgroup of the data collected in Munich (*n* = 1596) a logistic regression was conducted for the variables sex, age and nationality, as these data were readily available at the Munich site. The analysis revealed that males were significantly less frequently positive than females (OR (95%CI) = 0.17 (0.03–0.60) (Logistic regression), − 0.17 (0.02–0.74) (Fisher Exact)). Similarly, people with Brazilian/Bolivian nationality were significantly more often having a positive test result than other nationalities (OR (95%CI) = 273.48 (51.68–5059.88) (Logistic regression), 264.91(37.35–10,678.77) (Fisher Exact). Finally, for every unit increase in age the probability of being positive to the test significantly increased by 1.03 (1.01–1.06).

## Discussion

This study represents the first comprehensive description of screening, detection, diagnosis, and treatment of CD in Germany. Of the five centres identified to perform CD testing for routine patient care, only the centres in Hamburg and in Munich offered two independent serological assays during the complete time period 2000–2018 as required by WHO for diagnosis. The centre in Bonn offered two independent tests during the interval of 2000–2010 and thus most German centres were only able to perform screening measures, if current recommendations are considered [16]. Taking into consideration that *T. cruzi* infections were relatively new to Germany in the beginning of the 2000’s as detailed in the introduction section, laboratory diagnosis was pioneering work in the country. Nevertheless, it has to be stated that 20 years later there is still a precarious lack of standardized policies in this regard. Previous studies named the absence of clear recommendations as well as standardisation in respect of screening, detection, diagnosis, and treatment as an important risk factor for inadequate care of individuals at risk [21, 22].

The data presented here is likely patchy; some smaller and/or private laboratories performing commercially available CD diagnostics during patient care might have been missed in this study. Furthermore, the identification of individual patients could not be performed with absolute reliability due to the pseudonymised nature of retrieved data. Although data was cleaned and controlled for quality, likely not all duplicate cases could be detected. For diverse reasons (including water damages on as well as disposal of paper-based patient records or technical problems with aged laboratory information systems leading to data loss) not all data could be retrieved. This proves the difficulty of digitalization and data availability/storage. In Germany, a total of *n* = 127,615 migrants from endemic countries have been registered in 2018 [11]. However, when reflecting on prevalence and testing frequencies, a presumably significant number of undocumented migrants as well as migrants with European citizenship that originated from endemic countries have to be taken into account. According to the available data in this study, a total of *n* = 5991 tested patients could be identified in Germany during the time period 2000–2018, whereas only *n* = 130 (16.0%) of tested individuals with known nationality were from endemic countries; thus, all numbers presented have to be interpreted carefully.

Due to missing information, it is difficult to draw conclusions on the distribution of CD testing performed between German expatriates/travellers, people originating from LA countries with German nationality, or LA migrants. A previous study has calculated an overall prevalence of 4.2% among LA migrants in Europe [8]. When taking into account the number of registered migrants, undocumented migrants as well as LA migrants with European citizenship in Germany, and signs that potentially most tests in this study have been performed on German expatriates/travellers, access to screening, detection, diagnosis, and eventually medical care appears to be alarmingly low for people at highest risk for CD. This has also been mentioned as a problem in studies performed in other European countries. The definition of “migrant,” which is not universally defined, and the lack of information about participants’ country of birth negatively influences the collection of data regarding CD in European countries. In order to assess the actual prevalence of CD, the number of undocumented migrants and the number of LA migrants with European citizenship has to be included. Most European countries lack databases that facilitate the viewing of such information [23].

The cumulative quantity of tests performed that could be retrieved for this study showed an increase over time, especially during the last 5 years. A distinct increase in tests performed in the years 2014–2018 might possibly be explained by migratory patterns of LA migrants to Germany: an increase of immigration could also be observed during this time period, specifically for females [24]. This could partly be caused by the economic crisis in Spain 2010–2015. Spain is the European country with the largest proportion of LA migrants and the largest number of reported CD cases [9]. With this, it plays a major role in the LA migrant flow and experienced increased emigration during the years of the crisis [25]. According to the national statistical institute in Spain, there was an increased number of LA migrants moving to Germany with a peak in 2013 and a total of *n* = 25,397 LA emigrants from Spain respectively [26]. However, this dynamic might partly be caused by the fact that older data were more difficult to be retrieved at some of the centres in this study leading to potential loss of data. Additionally, increased activities to raise awareness and to actively detect CD in Germany could also have contributed to the increase [12]. Similar trends can be observed, when comparing mean ages of the LA migrant population in Germany with the mean ages of the individuals included in this study. In our study, mean age was 39.1 years (SD = 16.7) compared to the mean age of the German population being 45.9 in 2019 [27].

A total of *n* = 81 CD patients was identified in this study. Again, this number has to be interpreted carefully; although it would correspond to a CD prevalence of 1.4% among all individuals tested, it includes already previously diagnosed CD patients (*n* = 28/43, 65.1%; denominator is patients for whom data on initial diagnosis was given) actively seeking follow-up as well as many German expatriates/travellers. It is therefore difficult to compare our results to previous European studies [28–31]. Of all *n* = 130 individuals tested that are known to be LA migrants, a total of *n* = 35 (26.9%) tested positive for *T. cruzi* infection. Other studies performed in Europe, focused exclusively on the LA population and on different subgroups. For instance, in a study performed in Switzerland a prevalence of 12.8% CD was found, however the screening only focused on Latin American adults [32].

Additional data from the risk factor analysis for the Munich data demonstrated that men were less likely to be positive for Chagas when compared to women. Also, the risk for being positive increases with increased age and nationality being in the Chagas hotspots such as Brazil and Bolivia compared to other places. Many CD studies suggest different results regarding the association between CD and sex. Nevertheless, it has been shown, that women are not necessarily more prone to be infected with CD, but that they are screened more often and hence CD infections could potentially be found more often in women than in men. This mainly occurs because of the different screening programs that have been implemented for women at a fertile age or for pregnant women, both in LA countries and in European countries like Switzerland, Spain, and Italy [33]. In regards to the association of age with CD, other studies have suggested that CD vectorial transmission in endemic countries has decreased over the past 20 years. Individuals that are now tested positive in European countries were probably infected a long time ago before migrating and are now becoming symptomatic, or have been previously diagnosed in their home countries [31]. The association with nationality and CD, has been demonstrated in many studies. For instance, based on seropositivity studies conducted on LA migrants in Europe, Bolivians had the highest prevalence of CD (18.2%) when compared with other endemic countries like El Salvador, or Argentina [34]. This is also dependent on the current rates of infection in the endemic countries, for which Bolivia has been shown to be higher [1].

Pregnancy was not mentioned as a reason for testing, even though *n* = 35/80 (43.8%) of detected patients were females of childbearing age. Of the *n* = 29 patients, information was available concerning etiologic treatment: only *n* = 13 (44.8%) of these received either benznidazole or nifurtimox. Reasons for not etiologically treating patients mentioned by the responsible physicians were frequently not in line with contemporary recommendations. Previous studies estimated that globally only 1% of people affected with CD receive etiological treatment [35]. Even in a country like Germany with substantial resources in the health care sector, these estimations don’t seem to be contradicted. BNZ and nifurtimox are neither registered nor available in Germany, hence they have to be obtained elsewhere (e.g. WHO) and used unlicensed. This certainly could contribute to low deployment rates.

In Germany, blood donors currently are being screened for CD by asking whether they are suffering from a parasitic infection (and sometimes specifically for CD) and some might be excluded when originating from for malaria endemic areas [12, 36]. At the same time, it should be considered that 94–96% of *T. cruzi* infected individuals from endemic countries are unaware of their infection status [9, 37]. In comparison, countries like Spain or USA have implemented serological screening measures in the transfusional/transplantational sector by law [1]. There is no routine screening performed on pregnant women from endemic countries [9, 37]. It was already stated by WHO in 2009 that women of fertile age should be a target group for CD screening in order to prevent vertical transmission of *T. cruzi* infections [7]. All in all, prevention of non-vectorial transmission of *T. cruzi* infection seems ineffective in Germany. These are important points to consider for future policy changes.

Several previous studies looked at personal and structural barriers to access of healthcare in Germany and in Europe, also with regard to CD [12, 38]. As an example, migrants from CD endemic countries without a valid residence permit and without health insurance are known to have the highest CD prevalence rates while their access to healthcare in Germany is outmost limited [12].

Several limitations of retrospective observation have already been mentioned —notably the absence of clear criteria for testing, differences in diagnostic algorithms across the different institutes, the large amount of missing information, and the non-respect of the full diagnosis algorithm. Despite the aforementioned limitations, this study highlights that even though Germany performed a pioneering implementation of screening, detection, diagnosis, and treatment of *T. cruzi* infections in regard to the knowledge that was available during the first period of the nearly two decades included in this study, currently there seems to be only a small percentage of individuals at risk of *T. cruzi* infection receiving adequate care. Previous studies show that a lack of guidelines is an important risk factor for inadequate care [21, 22] and regard the implementation of a screening programme for *T. cruzi* infection among Latin American adults living in Europe a cost-effective strategy [10]. A prospective survey will be needed in the future to have a better estimation of the prevalence of CD in Germany. This study was rather aiming at exploring past and current practices in terms of screening, diagnosis, and treatment of CD in Germany taking into account that the time period that was included was only the beginning in the development of knowledge of CD in this country. It is clear that the way forward should include an increase in training for certain groups of physicians (e.g. general practitioners, gynaecologists, paediatricians, and cardiologists) and Latin American communities, as well as the involvement of CD patient groups. Information campaigns should be developed, in order to directly approach members of the Latin American communities —for example in cultural events and via NGO’s that are working with migrants and/or churches. In addition, easy and reliable access to healthcare especially needs to be facilitated for undocumented migrants in order to render control measures effective. Additionally, systematic screening of pregnant women who come from CD endemic countries should be implemented, following the response in countries like Spain, Italy, and Switzerland. Screening should also be implemented in blood donors, for example in France a systematic screening of all blood donors that were born or lived in CD endemic countries was implemented [39]; policies that include this kind of screening could be cost-effective [10]. Due to the previously mentioned reasons, existing efforts to create German guidelines on screening, detection, diagnosis, and treatment of *T. cruzi* infection should be materialized, with the ultimate goal to improve access and quality of care regarding this highly neglected disease.

## Conclusions

Data on Chagas disease is patchy in Germany, and systematic screening of risk groups or notification mechanisms are lacking. Over the course of the studied time period, the different included centers disposed of diverging diagnostic means, rendering comparison of the collected data difficult. Coherent national guidelines are urgently missing for screening, diagnosis and treatment of this potentially fatal parasitic infection.

## Supplementary information

**Additional file 1:** **S1 File.** English translation of the questionnaire for physicians treating patients with Chagas disease.

**Additional file 2:** **S2 Table.** Detailed description of tests and procedures in each institution.

## Data Availability

The datasets generated and/or analysed during the current study are not publicly available due to data ownership by the different involved institutions, but are available from the corresponding author on reasonable request.

## Abbreviations

BNZ: Benznidazole
DTG: German Society for Tropical Medicine and Global Health
ELISA: Enzyme Linked Immunosorbent Assay
IFT: Immunofluorescence Tests
PCR: Polymerase Chain Reaction
RDT: Rapid Diagnostic Testing
