# Supplementary material for: Describing nearly two decades of Chagas disease in Germany and the lessons learned: a retrospective study on screening, detection, diagnosis, and treatment of Trypanosoma cruzi infection from 2000 – 2018
Source: BMC Infect Dis. 2020 Dec 3;20:919. doi: 10.1186/s12879-020-05600-8 (PMC7713040; doi:10.1186/s12879-020-05600-8)
Supplement: Supplementary file 1 — Additional file 1: S1 File. English translation of the questionnaire for physicians treating patients with Chagas disease. [file 12879_2020_5600_MOESM1_ESM.docx]

**Supplement 1: English translation of the questionnaire for physicians treating patients with Chagas disease**

Please read the following questions carefully. After answering all questions, sign the questionnaire, and add the date. Please send the questionnaire inside of the adjacent pre-paid envelope to the following address:

XXX

Thank you for your contribution for the improvement of the care of Chagas- patients in Germany!

# Date of birth and gender:

**Date of test:**

What nationality(ies) did the patient have?

What was the reason for testing?

If the reason for testing was travelling outside of Germany, what was the travel history (countries, duration, month(s), and year(s))?

Did the patient have any symptoms at the time of testing? If yes, what were the symptoms and how long were these present?

Was the patient treated with Benznidazol or Nifurtimox? If yes, describe the treatment procedure (medication, dosage, duration)?

If the patient was treated with Benznidazol or Nifurtimox, where and how was the medication obtained?

If the patient was treated with Benznidazol or Nifurtimox, was the therapy interrupted? If yes, why?

f the patient was treated with Benznidazol or Nifurtimox, were there any complications during the treatment? If yes, what were the complications?

If the patient was not treated with Benznidazol or Nifurtimox, what was the reason for this decision?

Was an ECG performed? If yes, what results did it yield?

Was an echocardiography performed? If yes, what results did it yield?

Were there any additional diagnostic tools/ therapies performed? If yes, what type of diagnostic tools/therapies and by whom were these performed (e.g. gastroenterologist, cardiologist, etc.)?

Is the patient regularly supervised (e.g. ECG examination every 6-12 months)? If yes, how? If not, what was the reason for this decision?

Do you speak Spanish or Portuguese?

City, date Signature (and stamp)
