# Supplementary material for: Describing nearly two decades of Chagas disease in Germany and the lessons learned: a retrospective study on screening, detection, diagnosis, and treatment of Trypanosoma cruzi infection from 2000 – 2018
Source: BMC Infect Dis. 2020 Dec 3;20:919. doi: 10.1186/s12879-020-05600-8 (PMC7713040; doi:10.1186/s12879-020-05600-8)
Supplement: Supplementary file 2 — Additional file 2: S2 Table. Detailed description of tests and procedures in each institution. [file 12879_2020_5600_MOESM2_ESM.docx]

**Supplement 2: Detailed description of tests and procedures in each institution**

| Institute of Tropical Medicine and International Health, Charité – Universitätsmedizin, Berlin | |
| --- | --- |
| Type of tests | **Time period (years)** |
| in-house ELISA | 2000-2018 |
| in-house IIF | 2000-2010 |
| Description | |
| - Reference values for ELISA: <5 = negative, 5-10 = borderline, >10 = positive - Reference values for IIF: <1:20 = negative, ≥1:80 = positive - Patient samples with a reactive test result were either previously known to be positive or sent to other laboratories for confirmation | |
| Institute of Medical Microbiology, Immunology and Parasitology, University Hospital Bonn | |
| Type of tests | **Time period (years)** |
| commercial ELISA (bioelisa CHAGAS biokit) | 2011-2018 |
| in-house IFT | 2000-2010 |
| commercial ID- Chagas antibody test (PaGIA) ^[1]^ | 2000-2010 |
| Description | |
| - Positive results that came in before 2011 were confirmed by performing the above mentioned ELISA retrospectively in the year 2019 using retained samples - Reference values for ELISA: <0.9 = negative, ≥0.9-<1 = borderline, ≥ 1 = positive^[2]^ - Reference values for IFT: <1:16 = negative, ≥1:16-<1:64 = borderline, ≥1:64 = positive - IFT of Dipl.- Biol. Charles Soukou (T. cruzi Chilean strain), reference values ≥ 1:40 = positive; 1:20 = borderline; < 1:20 = negative | |
| Bernhard Nocht Institute for Tropical Medicine, Hamburg | |
| Type of tests | **Time period (years)** |
| in-house ELISA | 2000-2018 |
| in-house IFA | 2000-2018 |
| PCR^[3]^ | 2000-2018 |
| Description | |
| - Reference values for ELISA: <0.1 = negative, ≥0.1-≤0.2 = weak positive, ≥0.2 = positive - IFA reference value is <1:20 - Positive PCR results were followed by sequencing of the respective PCR product | |
| Division of Infectious Diseases and Tropical Medicine, University Hospital, LMU Munich | |
| Type of tests | **Time period (years)** |
| in-house ELISA | 2000-2018 |
| in-house IFAT | 2000-2018 |
| in-house PCR^[4]^ | 2013-2018 |
| Description | |
| - For the ELISA, the reference values are, >10 = positive; 10-14 = borderline - For the IFAT the reference values are 1:64 = borderline; >1:64 = positive - A conventional PCR was performed and if positive followed by an independent, commercially available qPCR ^[4]^ | |
| Institute of Tropical Medicine, Medical Department, University Hospital Tübingen | |
| Type of tests | **Time period (years)** |
| in-house IIFT | 2009-2018 |
| Description | |
| - For the IIFT, the reference value is >1:64 = positive - Patient samples with a reactive test result were either previously known to be positive or sent to other laboratories for confirmation | |

1. Rabello A, Luquetti AO, Moreira EF, Gadelha M de F, dos Santos JA, de Melo L, et al. Serodiagnosis of Trypanosoma cruzi infection using the new particle gel immunoassay--ID-PaGIA Chagas. Mem Inst Oswaldo Cruz. 1999 Feb;94(1):77–82. doi: [10.1590/s0074-02761999000100016](https://doi.org/10.1590/s0074-02761999000100016).
2. World Health Organization [Internet]. Anti-trypanosoma cruzi assays: operational characteristics, report 1; c2010 [cited 2019 Aug]. Available from: <https://apps.who.int/iris/handle/10665/75844>
3. Wincker P, Britto C, Pereira JB, Cardoso MA, Oelemann W, Morel CM. Use of a simplified polymerase chain reaction procedure to detect Trypanosoma cruzi in blood samples from chronic chagasic patients in a rural endemic area. Am J Trop Med Hyg. 1994 Dec;51(6):771–7. doi:10.4269/ajtmh.1994.51.771
4. Seiringer P, Pritsch M, Flores-Chavez M, Marchisio E, Helfrich K, Mengele C, et al. Comparison of four PCR methods for efficient detection of Trypanosoma cruzi in routine diagnostics. Diagn Microbiol Infect Dis. 2017 Jul;88: 225–232. doi:10.1016/j.diagmicrobio.2017.04.003.
